# Anthropogenic Resource Subsidies Determine Space Use by Australian Arid Zone Dingoes: An Improved Resource Selection Modelling Approach

**DOI:** 10.1371/journal.pone.0063931

**Published:** 2013-05-30

**Authors:** Thomas M. Newsome, Guy-Anthony Ballard, Christopher R. Dickman, Peter J. S. Fleming, Chris Howden

**Affiliations:** 1 Institute of Wildlife Research, School of Biological Sciences, Heydon-Laurence Building, University of Sydney, New South Wales, Australia; 2 Invasive Animals Co-operative Research Centre, University of Canberra, Canberra, Australian Capital Territory, Australia; 3 Vertebrate Pest Research Unit, NSW Department of Primary Industries, University of New England, Armidale, New South Wales, Australia; 4 Vertebrate Pest Research Unit, NSW Department of Primary Industries, Orange, New South Wales, Australia; 5 Tricky Solutions, Sydney, Australia; The University of Wollongong, Australia

## Abstract

Dingoes (*Canis lupus dingo*) were introduced to Australia and became feral at least 4,000 years ago. We hypothesized that dingoes, being of domestic origin, would be adaptable to anthropogenic resource subsidies and that their space use would be affected by the dispersion of those resources. We tested this by analyzing Resource Selection Functions (RSFs) developed from GPS fixes (locations) of dingoes in arid central Australia. Using Generalized Linear Mixed-effect Models (GLMMs), we investigated resource relationships for dingoes that had access to abundant food near mine facilities, and for those that did not. From these models, we predicted the probability of dingo occurrence in relation to anthropogenic resource subsidies and other habitat characteristics over ∼ 18,000 km^2^. Very small standard errors and subsequent pervasively high *P*-values of results will become more important as the size of data sets, such as our GPS tracking logs, increases. Therefore, we also investigated methods to minimize the effects of serial and spatio-temporal correlation among samples and unbalanced study designs. Using GLMMs, we accounted for some of the correlation structure of GPS animal tracking data; however, parameter standard errors remained very small and all predictors were highly significant. Consequently, we developed an alternative approach that allowed us to review effect sizes at different spatial scales and determine which predictors were sufficiently ecologically meaningful to include in final RSF models. We determined that the most important predictor for dingo occurrence around mine sites was distance to the refuse facility. Away from mine sites, close proximity to human-provided watering points was predictive of dingo dispersion as were other landscape factors including palaeochannels, rocky rises and elevated drainage depressions. Our models demonstrate that anthropogenically supplemented food and water can alter dingo-resource relationships. The spatial distribution of such resources is therefore critical for the conservation and management of dingoes and other top predators.

## Introduction

Dingoes (*Canis lupus dingo*) most likely arrived in Australia on boats from Asia at least 4,000 years ago [1]–[3]. Sometime after their introduction and adoption by indigenous Australians, dingoes became feral and have since occupied nearly every terrestrial habitat on the mainland [4]. Dingoes, like free-roaming dogs elsewhere, interact with humans and the water and food resources they provide either purposely or accidentally though refuse and artificial water points [5]. Given dingoes’ anthropocentricity, we sought to clarify habitat use by dingoes in the presence of anthropogenic resource subsidies. This was best achieved in a region where human activity was focal and by analyzing dingo home-range data.

The home range of an animal or group of conspecific animals is often described by a multifaceted polygon that contains all the movements the animals need to attain resources for survival and reproduction [6]. Most animals do not traverse their home range using random walks. Instead, their movements typically reflect heterogeneous dispersion of resources across the landscape. Over the last decade, we have greatly improved data collection about the movements of medium to large sized animals (>1 kg) through relatively cheap Global Positioning System (GPS) technology [7]. This in turn has stimulated detailed investigations into how such species use space and resources, as well as the development of powerful analytical techniques to better quantify space–resource interactions.

Arguably the most popular technique for quantifying the relative use of habitat is Resource Selection Function (RSF) modelling [8]. An RSF is defined as any function that is proportional to the probability of an animal using a particular resource [9]. Resource selection modelling provides spatially explicit predictive models for animal occurrence by comparing habitat characteristics at any two of the following three types of sites: those that are used by animals; those that are unused; and those that are potentially available [10]. This approach has been applied to a wide variety of species including brown bears (*Ursus arctos*) [11], [12], moose (*Alces alces*) [13], mountain caribou (*Rangifer tarandus caribou*) [14], and rufous bristlebirds (*Dasyornis broadbenti*) [15], among others.

Despite the ability of RSF models to provide useful analytical data, no attempt has been made to develop a RSF for dingoes, or any other mammalian predator in Australia, despite an overwhelming amount of data being collected on their space use (e.g. [16]–[19]). One possible reason for this is the inherent difficulties involved in developing a RSF from large datasets. For example, compared with traditional VHF radio-tags, GPS units have the capacity to provide many regular fixes throughout the day for months, with the deployment period depending on frequency of fixes and battery life, which in turn is set by the weight of collar that animals can carry without affecting their activity patterns. While this allows collection of diurnal and nocturnal space use data without undue disturbance to target animals, it also challenges the usual assumption of independence that underlies RSF models [20]. To overcome this, data are sometimes removed on the assumption that an increased time-lag between fixes also increases independence [21]. However, as noted for studies employing more traditional VHF radio-tracking, *post hoc* censoring of fixes defeats the advantages of employing GPS technology [18] and collecting large and more informative datasets [11].

There is debate among specialists about suitable methods for analysis of such autocorrelated data [22]. Here, we use the second of the suggested methods of Fieberg et al. [22] which is a potentially powerful approach to generate a RSF by using Generalized Linear Mixed-effect Models (GLMMs) [10] that accommodate hierarchical correlation structures. Although the use–availability sampling design of GLMMs is dependent on the sampling rates of used and available points and is computationally demanding [21], [22], GLMMs allow predictive models of individual and group space use from serially correlated data. Sequential GPS fixes constitute such data (under the assumption of random effects within individuals causing correlations among the repeated measures (i.e. fixes) (see Fieberg et al. [22] for discussion of applicability of mixed-effects models to clustered used and available points)). Hence, to advance our understanding of anthropogenic influences on dingoes’ use of space and to develop predictive models of resource selection, we explored the use of GLMMs with a large data set of fixes from GPS-collared dingoes.

Our primary objective in developing the RSF was to determine what predictors of occurrence, including anthropogenic resources, describe dingo space use in the Tanami Desert of central Australia. In that remote, arid region there are few focal anthropogenic resource subsidies provided by mining and pastoral industries; any differences between the home ranges used by anthropocentric dingoes and others are likely maximized there. From an analytical perspective, we also provide commentary on the capacity of GLMMs to develop RSFs from large data sets of GPS fixes. In doing so, we advance an alternative methodology that uses effect sizes, not just *P*-values, to determine which predictors to include when selecting robust final models of probability of dingo occurrence in different habitats. This is particularly important for GPS tracking data since, if only *P*-values are used (the more traditional approach), the sheer number of fixes means that even if serial and spatio-temporal correlation is correctly modelled, standard errors and *P*-values will be very small and will lead to the ubiquitous statistical significance of all predictors used in the models.

## Materials and Methods

### Study Region and Resource Availability

**Figure 1:**
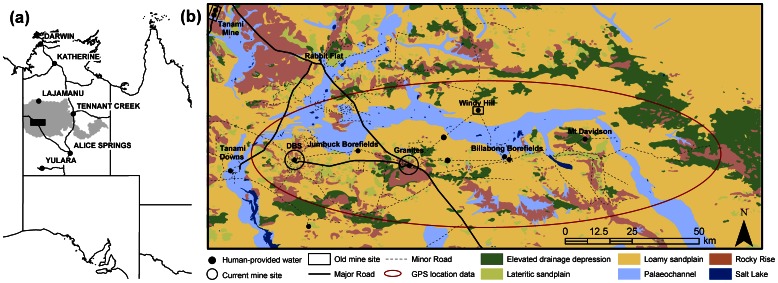
Location of the study region. (a) Study region (box), Tanami Desert (grey), in relation to major towns and roads in central Australia, and (b) study region where resource selection function modelling was undertaken. The general area where GPS fixes were retrieved is denoted by the red oval. Land-units are based on the regolith units of Wilford and Butrovski [50] and land-units of Domahidy [51].

The study region, covering approximately 18,000 km^2^ in the western portion of central Australia’s Tanami Desert (130° 18′ E, 20° 30′ S), was delimited by the perimeter of all GPS fixes that were obtained from collars fitted to dingoes between April 2008 and April 2010 (Figure 1). Land-use in the study region includes gold mining operations and pastoral activities. Mining operations are located at The Granites and Dead Bullock Soak (DBS). Disused gold mines with open-cut pits and no human occupation are located at Windy Hill and Tanami Mine, and cattle are kept on Tanami Downs. At the time of this study, there were 13 human-provided watering points permanently available to dingoes (Figure 1). Also available to dingoes were large quantities of food scraps within refuse facilities at The Granites and to a lesser extent at DBS. A roadhouse is located at Rabbit Flat and a farm house is occupied by pastoral workers on Tanami Downs. A series of major and minor tracks associated with both pastoral and mining operations exists throughout the study region (Figure 1).

There are no large hills in the study region, and digital elevation models [23] indicate an elevation profile between 225 m and 475 m above sea level. Lower-lying areas are associated generally with drainage depressions, salt lakes and palaeochannels. Wildfires occurred at scattered sites throughout the study region in 2007, resulting in vegetation of several age classes [unpublished data]. No major fires occurred during the present study.

### Study Animals and Telemetry Data

One hundred and eleven dingoes were live captured and released between April 2008 and April 2010 and collars housing a GPS data logger and a VHF transmitter (Sirtrack, Havelock North, New Zealand and Bluesky Telemetry, Aberfeldy, Scotland) were fitted to a sample of 23 adults. Both male and female dingoes were collared, but only if they weighed more than 20 times the weight of the collar. Collar weight also limited the sampling period so, to ensure that dingoes were tracked under all seasonal conditions, collaring was staggered throughout the study period. In doing so, we sampled dingoes in as many areas as possible along a latitudinal transect between DBS and Mt Davidson (Figure 1) whilst ensuring replicates of both males and females in the sampled areas where GPS fixes (locations logged and stored as longitude-latitude co-ordinates) had been recovered from retrieved collars. The GPS unit on every collar was programmed to estimate a fix each hour, with sampling rates based on battery-life calculations. Seven collars suffered mechanical failures and did not return any data, and three were not found, but the remainder logged GPS information for up to 10 months at a time. Data from the collars included an Horizontal Dilution Of Position (HDOP) value as well as the number of satellites used to calculate each fix. The HDOP was used to determine a Maximum Allowable Error (MAE) of fix accuracy by multiplying the HDOP value by the accuracy of the GPS device, which was 2.5 m [24]. Fixes with an HDOP value >8 were excluded to balance the number of usable fixes against positional accuracy, yielding a MAE of 40 m.

### Digital Environmental Data Sources

Explanatory/predictor variables (*italicized* below) that were thought to influence landscape-level distribution of dingoes were derived from 16 Geographic Information System (GIS) layers (Table 1). Six major *land-units* were used to broadly characterize landscape types (Figure 1). A measure of *land cover* was calculated from the mean values of 16–day 250 m Enhanced Vegetation Index (EVI) composites, taken over the study period, from the TERRA Moderate Resolution Imaging Spectroradiometer [25]. The mean EVI value was calculated in ArcView v9.2 (Environmental Systems Research Institute Inc.) using cell statistic tools in the Spatial Analyst extension. For analysis and ease of interpretation, mean EVI values were normalized by scaling the mean to zero and standard deviation to one.

**Table 1:** Description and characteristics of environmental and human-associated predictor variables used to model the probability of occurrence of dingoes in the Tanami Desert.

Variable	Name	Code	Resolution (m)	Units	Data range
Land-unit	Elevated drainage depression	EDD	40	Category	0 or 1
	Lateritic sandplain	LASP	40	Category	0 or 1
	Loamy sandplain	LOSP	40	Category	0 or 1
	Palaeochannel	PAL	40	Category	0 or 1
	Rocky rise	RR	40	Category	0 or 1
	Salt lake	SL	40	Category	0 or 1
Landcover	Enhanced vegetation index	EVI	40	n/a	32–2601
Terrain	Elevation	ELEV	40	Meters	227–475
Human	Distance to road (minor)	ROADMIN	40	Meters	0–49 564
	Distance to road (major)	ROADMAJ	40	Meters	0–132 616
	Distance to mine (old)	MINEOLD	40	Meters	0–112 856
	Distance to mine (current)	MINECURR	40	Meters	0–137 556
	Distance to camps	CAMPS	40	Meters	0–136 618
Food resources	Distance to refuse facility (minor)	TIPMIN	40	Meters	0–171 744
	Distance to refuse facility (major)	TIPMAJ	40	Meters	0–137 977
Water resources	Distance to water	WATER	40	Meters	0–80 285

*Elevation* data were derived from the Shuttle Radar Topography Mission 90 m Digital Elevation Model [23]. Roads were classified into two categories based on traffic use and size. *Roads (major)* included the Tanami Highway, the bulk haul road between DBS and The Granites and the access route to Tanami Downs from Rabbit Flat (Figure 1). *Roads (minor)* included all other tracks (Figure 1). Road alignment was confirmed using GEODATA Topo 250K v3 data [26] in association with onsite verification. *Mine sites (old)* included disused old open-cut pits, while *mine sites (current)* included all operational areas where ore was being extracted. *Camps* included areas around mine workers’ sleeping quarters, mess areas and operational office areas. *Refuse facilities (major)* included areas where commercial quantities of human-provided food scraps and other domestic refuse are discarded, while *refuse facilities (minor)* included areas where household quantities of refuse are discarded. The *water* GIS-layer included all permanent human-provided water sources such as those around bores, leaks in pipelines and where natural water had accumulated at the base of old mine sites. All potential water sources were inspected regularly over the study period and only those that were permanent were included.

### Data Preparation

Following retrieval of data from radio-collars, GPS fixes were plotted in ArcView v9.2. After overlaying all digital data (Table 1) in the same geographic projection the distance (m) from GPS fixes to each continuous predictor was calculated using the distance between points and nearest neighbour option in Hawth’s tools [27]. The attribute scores for all other predictors were derived using spatial joins. The kernel density estimator was chosen as a measure of home range because this non-parametric method is particularly robust in estimating probability density distributions of any shape [28]. The 100% kernel estimator was used because this likely represented the outer extremes of movement and thus encompassed the total range of resources that were potentially available to each dingo. As opposed to the adaptive kernel, the fixed kernel is more stable for defining probability contours of greater than 80%, so we used the latter. Kernel estimates were calculated in *R* [29] in the package adehabitat v1.8.3 [30] using the function *kernelUD*. The level of smoothing was determined by the default *adhoc* method (i.e. a bivariate normal kernel) as this resulted in shapes that appeared to be biologically realistic. Kernel boundaries were re-projected in ArcView v9.2 and converted into a grid (raster). Cells inside each kernel estimate were set as 1 and those outside as 0. A cell size of 40 × 40 m was chosen as this was the largest MAE derived from the GPS fixes. The raster boundaries were set as the outer limits of the study region (Figure 1). This resulted in a grid with 2279 rows and 4848 columns (11 048 592 cells or pixels).

Separate rasters of all digital data were created using the same parameters as the kernel estimates with the exception that each pixel represented the distance (m) from its centre to a continuous predictor or categorical unit value. In the case of the GPS data each cell also corresponded to presence (1) or absence (0) of a GPS fix. To check for any errors, maps of each raster were assessed for outliers using colour coding. Kernel estimates of home ranges were also cross-tabulated with GPS frequency data. No errors were detected.

To create the available resource units for each dingo, pixels were randomly sampled from inside the boundaries of its estimated kernel home range. Available resource units were sampled randomly at a rate of five times the number of GPS fixes obtained for each dingo; there is little benefit in taking more than four or five controls per case [31]. Distances to continuous predictors and attribute values for available resource units were derived from a merged spreadsheet of all digital data. We checked for errors by randomly extracting rows from the merged file to ensure they were identical to the originals, and by making maps that were then compared with the originals using colour coding. No errors were detected.

### Resource Selection Modelling

We developed an RSF model following the methods proposed by Gillies et al. [10] of using GLMMs with a binomial family and logit link function as well as a random intercept (individual dingo). This modelling approach is analogous to Design III in Thomas and Taylor [32] and Manly et al. [20] in that we sampled resource use for each individual dingo and calculated resource availability from a randomly sampled area within animals’ home ranges [20]; this is also referred to as a case–control study. The approach of Gillies et al. [10] in using random intercepts for each dingo is particularly relevant to our study because there was unequal sampling intensity due to the death of three dingoes and incomplete data from two GPS collars. Fieberg et al. [33] and Fieberg et al. [22] recommend that GLMMs, rather than Generalised Estimating Equations (GEEs), be used for population-level response patterns, such as those of dingoes to anthropogenic resources and other parameters. Only the results from GLMMs with a random intercept were considered (but see discussion for further commentary about GEEs).

Because we collared dingoes in different areas throughout the study site with varying levels of human-provided resources, dingoes were grouped *post hoc* (following Newsome et al. [34]) into three categories based on inspection of the GPS data. These were: dingoes that almost wholly associated with the mine facilities (‘mine’); those that had no association with mine facilities and were focused around a single artificial watering point (‘away’); and those that moved between multiple-artificial watering points and the mine (‘intermediate’). In doing so we fully evaluated resource selection by groups of dingoes that utilized similar human-provided resources. Grouping all animals would not provide such detail to compare resource selection; however, to provide a reference, we modelled the data for all dingoes (‘all dogs’) as well. Replication was obtained at the level of the individually monitored dingoes in each model.

For each category of dingo all continuous predictor variables were screened to test for collinearity using pair-wise correlations in *R*. From each set of correlated predictors (i.e. *r* >0.8), the one that made most sense biologically was selected for inclusion in the model while others were removed [35]. Box-plots of *land-units* against each continuous predictor were also generated to determine if confounding factors prevented separation of observed effects due to *land-units* or predictors. The models were adjusted to account for such effects.

By selecting predictor variables based on the ecology of the dingo population, the output of logistic regressions yielded estimates proportional to the probability of resource use in a pixel or polygon [36]. Models could be contrasted by comparison of deviances [20] or other model selection techniques such as the Akaike Information Criterion [37]. However, when determining which predictors to include in the final model it was important to understand the effect size of predictors and not rely solely on statistical significance [38]. This was particularly important as we were dealing with samples where autocorrelation may have led to inappropriately small standard errors (and low *P*-values), as was evident in the results of our full and final GLMM. To understand the effect size, we converted the logistic regression parameters to odds ratios to review the effect size of each predictor on dingo occurrence. Data for the ‘mine’, ‘intermediate’ and ‘away’ categories were also modelled at three spatial scales to allow interpretation of the effect of scale on dingo behaviour and gain understanding of the effect size of each predictor. The spatial scales were 1 m (Scale 1), 1 km (Scale 2) and 10 km (Scale 3) for distance predictors, and 1 m (Scale 1), 10 m (Scale 2) and 100 m (Scale 3) for *elevation*. Data for ‘all dogs’ were modelled at the same spatial scale as the other models to provide a comparison.

Full model deviances (all predictors) were then compared to a null model (no predictors) with a chi-squared distribution test using the ‘anova()’ function in *R*. As a binomial distribution was modelled, the deviance was not expected to follow a chi-squared distribution; however, the difference in the deviance between the full and null models was expected to follow a chi-squared distribution [39]. For each model, the data were then split into pixels where a dingo was sampled (present) or not (absent). The average prediction was calculated for each category for all pixels. Here, better models will have a higher average prediction for pixels where dingoes were present and lower average prediction where they were not. We then converted the parameters of the logistic regressions to odds ratios by taking their exponentials. The odds ratio represents the odds of finding a dingo compared to the preceding integer (if the predictor is continuous) or a reference category (if the predictor is categorical). For example, if the odds ratio for distance to *water* is 0.5 and the scale of effect is 1 km, there is a 50% less chance of finding a dingo in a pixel that is 1 km away from water compared to a pixel that is 0 km away from water.

The odds ratio was plotted with 95% confidence intervals (CI) for each group of dingoes at the three spatial scales to compare the effect size of each predictor on the models. We considered that, if the effect size was still small at the Scale 2 (1 km) and Scale 3 (10 km) levels (i.e. with an odds ratio difference to 1 of ≤0.05), the predictor did not have a meaningful impact on dingo occurrence. The choice of scale size was based on the average hourly velocity or displacement of the studied dingoes, which ranged from 0.2–1.2 km/h. Hence, a predictor was excluded if it had no influence on dingo behaviour at a scale within the upper limits (1 km) of these movement rates as well as within a scale well beyond (10 km). The review of effect sizes was also used to choose an appropriate scale for a final model and predictive map. Effect sizes that were within a useable and meaningful range for prediction were chosen; i.e., where predicted probabilities of occurrence would change in a consistent gradient that was not too fine or too coarse over the chosen scale of effect, thus allowing ease of interpretation for management implications.

Parameter estimates from final RSF models were used to generate probability functions of the relative occurrence for each category of dingoes across the study region (i.e. 0–1 for every pixel in the study region). Due to limited computer memory *R* was unable to predict the entire 11 million(+) rows of data; thus, data were split into smaller pieces of 2 million rows using a World Programming Systems (WPS) module (World Programming Ltd). After making the predictions, files were imported into ENVI v4.8 (ITT Visual Information Solutions) and output with a header file containing the co-ordinates and properties of the grid. Image-to-image conversion used ArcView v9.2 to create a tagged image file format (.tiff). To display the.tiff (i.e. the final predictive map), the layer properties were set as stretched values with standard deviations and *n* = 2.

### Ethics Statement

This research was undertaken under the Animal Care and Ethics Authority O06/009 from Orange Animal Ethics Committee, clearance number A05020 from Charles Darwin University Animal Ethics Committee and permit number 33607 from Northern Territory Parks and Wildlife. The Central Land Council provided permit number CD004 for conducting research on Aboriginal Land. We adhered to all conditions related to the study.

## Results

### Data Overview

Data from 13 collared dingoes (four adult females, nine adult males) were obtained during the study period. Collars remained on dingoes on average for 198 days (range 33–300). Analysis of the spatial distribution of GPS fixes resulted in two male and two female dingoes being placed into the ‘mine’ category model (15 658 GPS fixes; range 1995–4626 per dingo), five males into the ‘intermediate’ category model (22 890 GPS fixes; range 2559–6752 per dingo), and two male and two female dingoes into the ‘away ‘category model (14 876 GPS fixes; range 713–6495 per dingo). Data were not recoverable from the remaining 10 collars.

### Correlations and Confounding Factors

Some predictors within the combined home range areas of each category of dingo were highly correlated (*r* >0.8) with distance to *refuse facility (major)*. In the ‘mine’ models these included *mine (current)*, *water* and *camps*. In the ‘intermediate’ models they included *road (major)*, *mine (current)*, *refuse facility (minor)* and *camps*. In the ‘away’ models they included *road (major)*, *mine (old)*, *mine (current)*, *refuse facility (minor)* and *camps*. In the ‘all dogs’ model they included *road (major)*, *mine (current)*, *refuse facility (minor)* and *camps*. All correlated predictors were removed from the analyses.

There were also several confounding factors in relation to the distribution of *land-units* and other predictors. In the ‘mine’ model, elevated drainage depressions tended to be further away from *road (major)*; palaeochannels tended to be further away from *roads (minor and major)* and *refuse facilities (major)*; and salt lakes tended to be further away from *road (minor)* and *refuse facilities (major)*. For all models, salt lakes, palaeochannels and loamy sand plains tended to be in areas of lower *elevation*. Rocky rises also occurred more frequently in areas of higher *elevation*.

### Spatial Scale and Effect Size – Review of Full Models

Chi-squared tests of the deviance of the full models to the null models indicated that the full models provided a significant fit to the data (all at *P*<0.001) (Table S1). There was very little difference between the average prediction for where dingoes were present or absent between models within the same category. However, the average prediction for dingo presence was much higher in the ‘mine’ models at all three spatial scales compared with the ‘intermediate’ and ‘away’ models. The average prediction for where dingoes were absent was also much lower in the ‘mine’ model compared with the ‘intermediate’ and ‘away’ models. This indicates that the ‘mine’ models had much better predictive capabilities compared with the other models (Table S1).

All chosen predictors had a significant impact on the full model parameters (at *P*<0.001) (Table S2). However, the effect size of continuous predictors was so small in the Scale 1 models as a dingo moved 1 unit away (i.e. 1 m) that no impact on dingo occurrence was detectable (Figure S1). At Scale 3, the effect size was so large that movement of one unit away from the resource meant the chance of seeing a dingo was very low (Figure S1). The Scale 2 model was therefore considered the most appropriate to develop a final RSF. However, several predictors in the ‘intermediate’, ‘away’ and ‘all dogs’ models still had very small effect sizes at the Scale 2 level. In the ‘intermediate’ model, distance to *mine (old*) and distance to *refuse facility (major)* had relatively small effect sizes compared with all other chosen predictors. The effect sizes of distance to *road (minor)* and *refuse facility (major)* were also relatively small for the ‘away’ model compared with all other chosen predictors (Figure S1). In the ‘all dogs’ model at the Scale 2 level, distance to *mine (old*), *road (minor)* and *refuse facility (major)* had relatively small effect sizes compared with all other chosen predictors (Figure S2). All the predictors with small effect sizes were therefore removed from the final models.

### Final Models

Chi-squared tests of deviance of the final models to null models indicated that they all provided a significant fit to the data (*P*<0.001) (Table 2). There was very little difference between the average predictions for where dingoes were present or absent between the full model and final models (Table 2). Hence, predictive power was much better in the ‘mine’ models compared with the ‘intermediate’, ‘away’ and ‘all dogs’ models.

**Table 2:** Chi-squared (χ^2^) tests of the deviance of the final logistic regression models to a null model and the mean average prediction for where dingoes were present (prediction–present) and absent (prediction–absent).

Model	Scale	?^2^	Df	*P*	Prediction – Present	Prediction –Absent
Mine	2	59464	11	***	0.79	0.04
Intermediate	2	13065	7	***	0.25	0.15
Away	2	10499	7	***	0.28	0.14
All dogs	2	52913	8	***	0.33	0.13

Df = degrees of freedom. *** = *P*<0.001.

**Figure 2:**
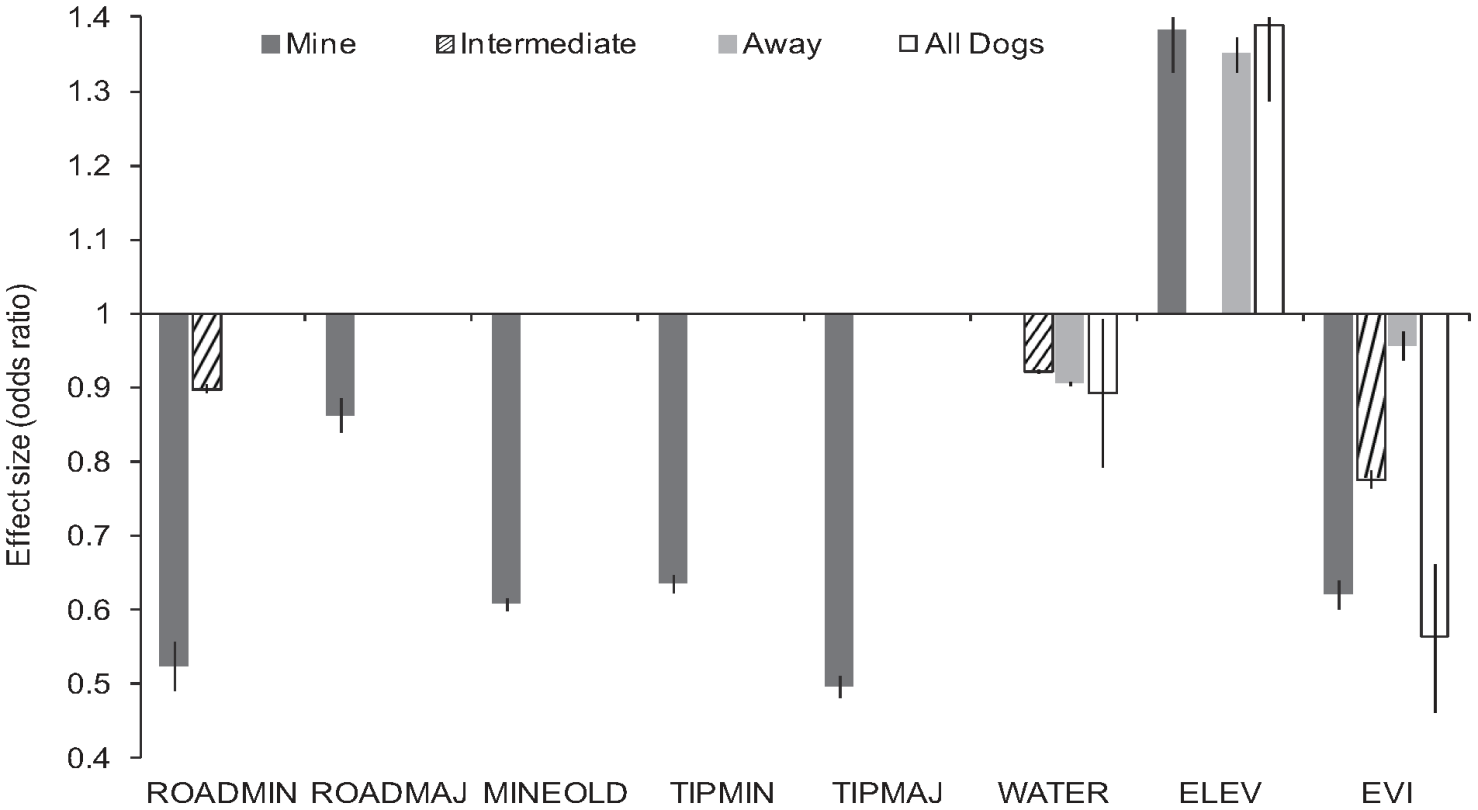
Effect size of continuous predictors on occurrence of dingoes in the Tanami Desert based on the results from the final generalized linear mixed model. Odds ratios are provided ±95% confidence intervals (CI). See Table 1 for X-axis acronyms.

All the chosen predictors in the final models had a significant impact on dingo occurrence (Table 3). However, the effect size of each predictor on dingo occurrence varied across each category of model. In particular, human-provided predictors, such as distance to *refuse facility (major)*, were much more important in the ‘mine’ model compared with the ‘intermediate’, ‘away’ and ‘all dogs’ models (Figure 2).

**Table 3:** Parameter estimates (β) and standard errors (SE) for predictors included in the final logistic regression models.

	Mine			Intermediate			Away			All dogs		
Predictor	β	SE	*P*	β	SE	*P*	β	SE	*P*	β	SE	*P*
Loamy sandplain (reference)	NA	NA	NA	NA	NA	NA	NA	NA	NA	NA	NA	NA
Elevated drainage depression	1.24	0.08	***	1.11	0.02	***	1.54	0.03	***	0.94	0.02	***
Lateritic sandplain	−0.31	0.06	***	0.45	0.03	***	0.52	0.04	***	0.13	0.02	***
Palaeochannel	1.59	0.13	***	1.20	0.02	***	0.94	0.03	***	1.38	0.02	***
Rocky rise	1.81	0.05	***	0.34	0.02	***	0.54	0.04	***	0.04	0.02	*
Salt lake	3.04	1.13	**	0.07	0.13	***	−0.38	0.16	*	−0.19	0.10	*
Enhanced vegetation index	−0.48	0.02	***	−0.25	0.01	***	−0.05	0.01	***	−0.58	0.01	***
Elevation	0.32	0.02	***	–	–	–	0.30	0.01	***	0.33	0.00	***
Distance to road (minor)	−0.65	0.03	***	−0.11	0.00	***	–	–	–	–	–	–
Distance to road (major)	−0.15	0.01	***	–	–	–	–	–	–	–	–	–
Distance to mine (old)	−0.50	0.01	***	–	–	–	–	–	–	–	–	–
Distance to mine (current)	–	–	–	–	–	–	–	–	–	–	–	–
Distance to camps	–	–	–	–	–	–	–	–	–	–	–	–
Distance to refuse facility (minor)	−0.70	0.01	***	–	–	–	–	–	–	–	–	–
Distance to refuse facility (major)	−0.45	0.01	***	–	–	–	–	–	–	–	–	–
Distance to water	–	–	–	−0.08	0.00	***	−0.10	0.00	***	−0.11	0.00	***

Scale was modelled at 1 km for distance predictors and 10 m for elevation. *** = *P*<0.001, ** = *P*<0.01, * = *P*<0.05.

### Predictive Maps

**Figure 3:**
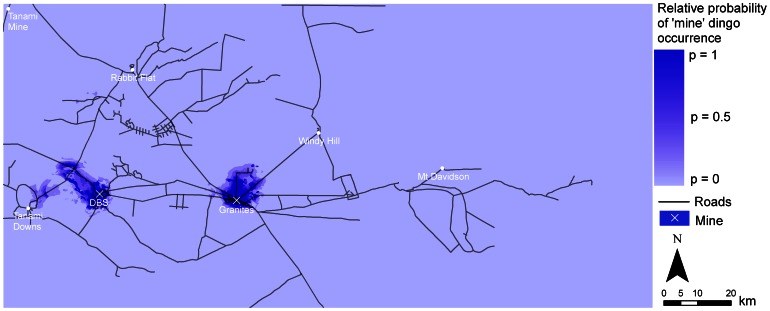
Predicted resource selection by ‘mine’ dingoes in the Tanami Desert at a scale of 1 km for distance predictors and 10 m for elevation.

**Figure 4:**
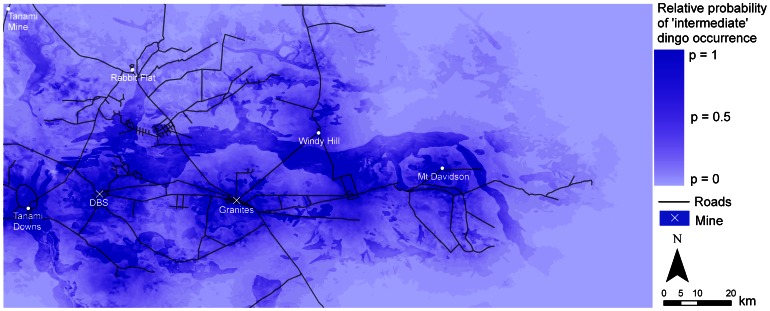
Predicted resource selection by ‘intermediate’ dingoes in the Tanami Desert at a scale of 1 km for distance predictors and 10 m for elevation.

**Figure 5:**
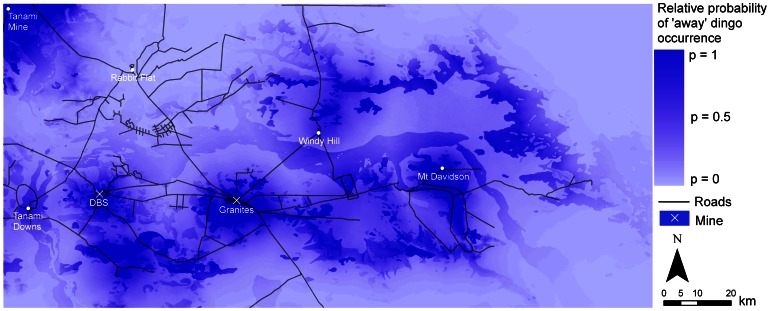
Predicted resource selection by ‘away’ dingoes in the Tanami Desert at a scale of 1 km for distance predictors and 10 m for elevation.

**Figure 6:**
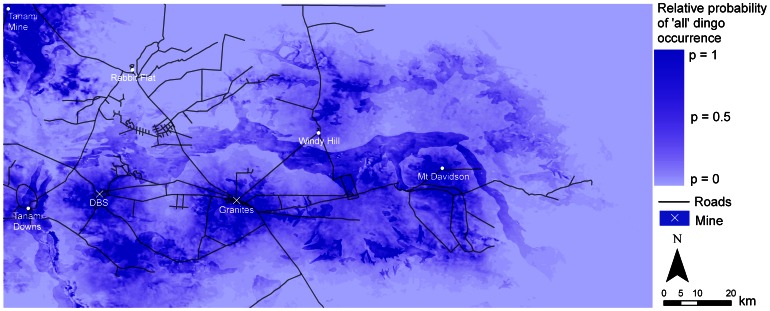
Predicted resource selection by ‘all’ dingoes in the Tanami Desert at a scale of 1 km for distance predictors and 10 m for elevation.

The variation in resource use across the four models was reflected in the predictive maps (Figures 3–6). In particular, due to the large effect size of human predictors in the ‘mine’ models, the probability of dingo occurrence was restricted to only a small area around the mine sites (Figure 3). In the ‘intermediate’, ‘away’ and ‘all dogs’ models, higher probabilities of occurrence were distributed across the study region, with higher values occurring within some land-unit boundaries. In the ‘intermediate’ model there was generally a higher probability of occurrence in the palaeochannel *land-unit* (Figure 4). In the ‘away’ and ‘all dogs’ model there was generally a higher probability of occurrence in the rocky rise and elevated drainage depression *land-unit* (Figures 5 and 6). In all cases, there were higher probabilities of occurrences in close proximity to *water* (Figures 4–6).

## Discussion

Our primary objective, from an ecological point of view, was to identify what predictors of occurrence influence dingo space use in the Tanami Desert. For dingoes that live primarily around mine sites, the most important predictor for dingoes (i.e. that with the largest effect size) was distance to *refuse facility (major)*. Human-provided food resources are therefore a key predictor for dingo occurrence around mine sites. For dingoes in the ‘intermediate’ and ‘away’ models, environmental variables had an important influence on the relative probability of occurrences of dingoes across the study region. In the ‘intermediate’ model there was generally a higher probability of occurrence in the palaeochannel *land-unit*, particularly in those close to *water*. In the ‘away’ models there was generally also a higher probability of occurrence in rocky rises and in the elevated drainage depression *land-units* adjacent to them.

The most important factors ordinarily affecting the distribution of dingoes are water, food and cover [4]. In theoretical frameworks describing the ecology of arid Australia [40], [41], the distribution of higher-order consumers is predicted to be restricted largely to, and reliant on, more productive refugia in the landscape such as calcrete and drainage substrates. In the Tanami Desert, the dingo has previously been shown to occur more often on fluvial substrates than on sand plains [42]. Lundie-Jenkins et al. [43] also found that drainage channels appeared to be important corridors for movement of dingoes at a site 15 km south-east of The Granites. Our data accord with the ideas of Stafford Smith and Morton [40] and Morton et al. [41] and previous track-based studies [42], [43]. However, our data highlight the potentially large impact of providing supplementary resources on dingo distribution. Particularly in the ‘mine’ model, there was no functional relationship between dingo occurrence and environmental variables that would otherwise be important in a system without anthropogenic resource subsidies.

Our findings, in relation to the effect of supplementary resources on dingo distribution, are pertinent to gaining an insight into how anthropogenic activity can influence dingo distribution. The overall importance of human-provided *water* for dingoes in the Tanami Desert is highlighted by the fact that there was only a 25–30% chance of finding an ‘intermediate’ or ‘away’ dingo in a pixel that was 10 km from *water*, compared to a pixel that was 0 km away. Dingoes generally drink water every day, about a litre in summer and half a litre in winter [44], but see Allen [17]. It is therefore unsurprising that *water* is a key driver of dingo space use. However, from a management point of view, this finding poses the question of whether or not dingoes would survive in the Tanami Desert without human-provided watering points. Given the high occurrence of the ‘away’ dingoes in rocky rises (Figure 1), it is possible that suitable substrates (in rock holes) could hold water following rainfall. During our study though, rainfall was below average at 219 mm and 263 mm in 2008 and 2009, respectively [45]. While this may have been enough to fill some rock holes for short periods, the timing of our study when rainfall was low perhaps increased the reliance of dingoes on human-provided water.

Robley et al. [18] noted that the ‘spatial scale at which wild dog management occurs needs to be reconsidered’. While this statement referred to the size of buffer zones that need to be applied in conventional programs for the poison baiting of wild dogs for livestock protection in south-eastern Australia, it applies equally to areas where the maintenance of dingo populations is the management objective. As demonstrated in our models, watering points were important predictors of dingo occurrence to the extent that as one moves up to 10 km away, the probability of dingo occurrence becomes relatively low (Figures 3–6). Hence, in arid Australia, where the availability of water is highly variable and largely dependent on rainfall, the spatial distribution of permanent watering points is a critical factor when considering the use of conservation reserves for dingoes [46]. *Water* in the Scale 3 model showed an exceptionally low odds ratio (<0.3) for the ‘intermediate’ and ‘away’ dingoes. This suggests that dingoes in the Tanami Desert require water resources to be not much more than 10 km apart, and certainly no more than 20 km apart (where the odds ratio of detecting a dingo drops to below 0.1).

Our study is the first to generate a RSF from GPS data for dingoes. Because our experimental design was unequally balanced (i.e. different numbers of fixes for each animal sampled) and excluding data from the models was not a preferred option, we adopted the analytical method of Gillies et al. [10]. In a review of this approach, Koper and Manseau [21] argued that it can be sensitive to incorrect variance-covariance and correlation specifications, with an internal correlation structure that could lead to biased standard errors. According to Koper and Manseau [21], Gillies et al. [10] stated that random effects can account for correlations from recording multiple fixes from each animal. However, the statement of Gillies et al. [10] in fact refers to clustering, or correlation amongst the animals. It does not refer to the autocorrelation that exists within each animal due to the close proximity of fixes.

In our models, the standard errors of the parameter estimates were very small (Table 3) for at least two reasons. First, a very large number of used and available data points was included (up to 100 000 per model), and secondly these points were assumed to be independent. The number of data points can have a very large influence on standard errors, even if the dependence issue is solved. If, for example, we had only used every 100^th^ data point in an attempt to ensure independence (gap between fixes would be approximately four days), this would have given up to 1000 data points for some models, which is enough for standard errors to still be very small. In consequence, the standard errors in our models were so small that even odds ratios that were close to one were statistically significant. It would have therefore been optimal to generate robust standard errors to compare to the model standard errors in this study. Generalized Estimating Equations (GEEs) can be used to fit robust standard errors [21], [22]. However, this process could not be completed in *R* using geepack v1.0-17 [47]–[49] with our large dataset. Even so, Gillies et al. [10] noted that the robust standard errors of GEEs may be biased towards animals with higher sample sizes, so this method may not have provided any more confidence in our models. Additionally, the problem of having predictors that were all statistically significant meant that traditional statistical methods for assessing likelihood, and hypothesis testing for model selection, were not helpful in determining the predictors to include in final models. By reviewing effect sizes at three different spatial scales we overcame this problem.

The issue of all predictors being significant is likely to arise with large datasets even if serial and spatiotemporal correlation is accounted for. Focusing efforts on overcoming issues related to data size is therefore likely to be more important than dealing with serial and spatiotemporal correlation. Hurlbert and Lombardi [38] stated that if sample sizes are too large, one may be “in danger” of getting very low *P*-values and establishing the sign and magnitude of even small effects with too much confidence. This issue of ‘Big Data’ or ‘Obese N’ has been overlooked in many reviews of approaches to RSF models because the focus is solely on autocorrelation (e.g. [22]). However, in our study, by analyzing the data at three spatial scales and reviewing the effect sizes, it was possible to identify which variables had meaningful effects on dingo occurrence. This in turn allowed the development of final models that included only these predictors. Reviewing effect sizes at different spatial scales is therefore one potential way to overcome the problem of having large datasets obtained from GPS tracking studies. This is a problem that will become more common as GPS tracking devices become more prevalent and data sizes increase.

## Supporting Information

Figure S1 **Effect size of continuous predictors of occurrence of dingoes in the (a) ‘mine’, (b) ‘intermediate’ and (c) ‘away’ categories based on the results from full generalized linear mixed models (GLMM) at three spatial scales in the Tanami Desert.** Odds ratios are provided ±95% confidence intervals (CI).* ELEV at Scale 3 in (a) is not shown as it had an odds ratio of 25.48 (95% Confidence Interval (CI) lower bound 16.95, CI upper bound 38.36); and in (c) it is not shown as it has an odds ratio of 6.56 (CI lower bound 5.38, CI upper bound 7.99). The spatial scales were 1 m (Scale 1), 1 km (Scale 2) and 10 km (Scale 3) for distance predictors, and 1 m (Scale 1), 10 m (Scale 2) and 100 m (Scale 3) for *elevation*.
(PDF)

Figure S2 **Effect size of continuous predictors of occurrence of dingoes in the ‘all dogs’ model based on the results from the full generalized linear mixed model (GLMM) at a spatial scale of 1 km (Scale 2) for distance predictors and 10 m (Scale 2) for** ***elevation*** **.** Odds ratios are provided ±95% confidence intervals (CI).
(PDF)

Table S1 **Chi-squared (χ^2^) tests of the deviance of the full models to a null model (no predictors) and mean average prediction for where dingoes were present (prediction – present) and absent (prediction – absent).** Df = degrees of freedom. *** = *P*<0.001.
(PDF)

Table S2 **Parameter estimates (β) and standard errors (SE) for continuous predictors included in the full models in the Tanami Desert.** Models were fitted with a random intercept following Gillies et al. [10]. *** = *P*<0.001.
(PDF)
