# Supplementary figures and images for: Anthropogenic Resource Subsidies Determine Space Use by Australian Arid Zone Dingoes: An Improved Resource Selection Modelling Approach
Source: PLoS One. 2013 May 30;8(5):e63931. doi: 10.1371/journal.pone.0063931 (PMC3667862; doi:10.1371/journal.pone.0063931)

**Figure S1**

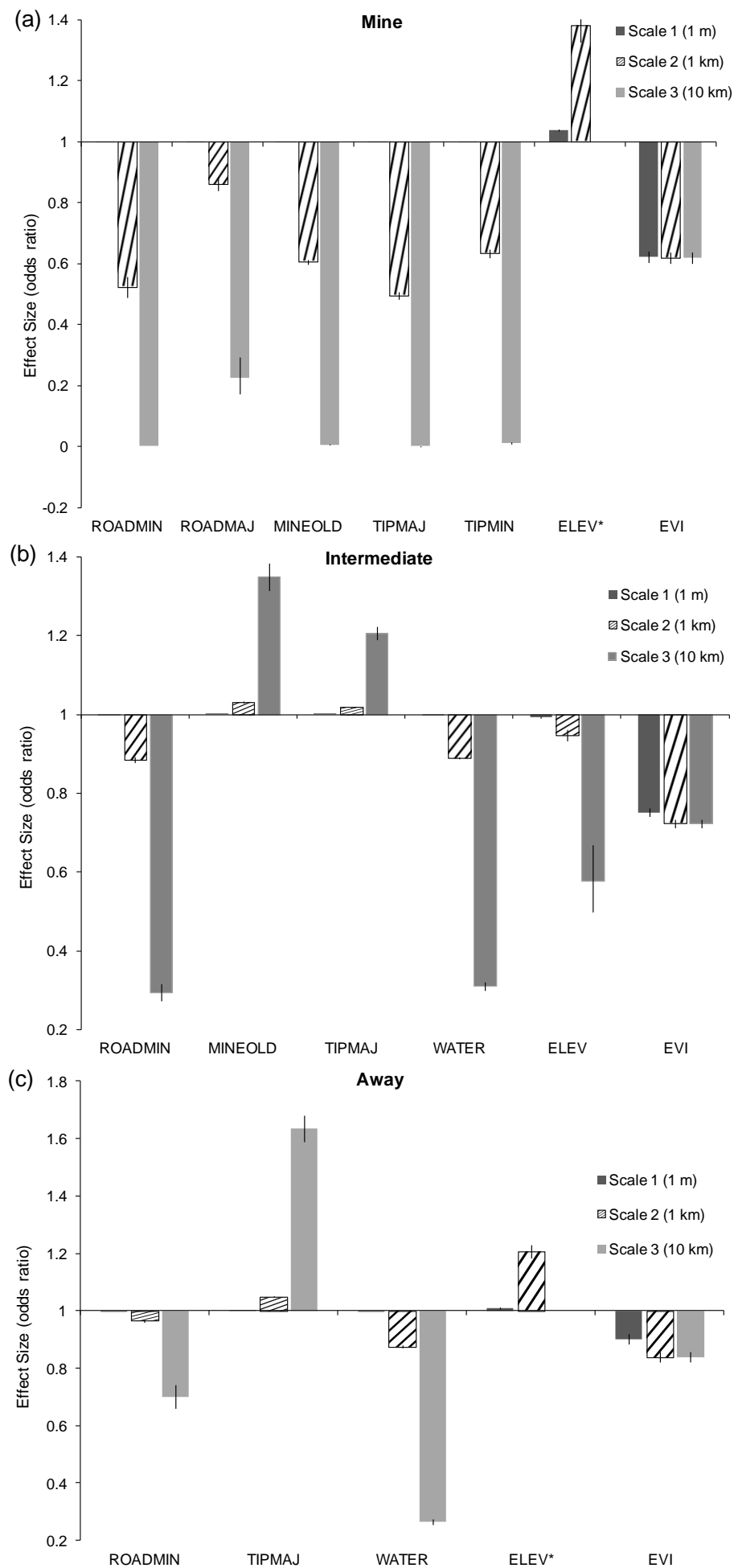

Supplement: Figure S1 — Effect size of continuous predictors of occurrence of dingoes in the (a) ‘mine’, (b) ‘intermediate’ and (c) ‘away’ categories based on the results from full generalized linear mixed models (GLMM) at three spatial scales in the Tanami Desert. Odds ratios are provided ±95% confidence intervals (CI).* ELEV at Scale 3 in (a) is not shown as it had an odds ratio of 25.48 (95% Confidence Interval (CI) lower bound 16.95, CI upper bound 38.36); and in (c) it is not shown as it has an odds ratio of 6.56 (CI lower bound 5.38, CI upper bound 7.99). The spatial scales were 1 m (Scale 1), 1 km (Scale 2) and 10 km (Scale 3) for distance predictors, and 1 m (Scale 1), 10 m (Scale 2) and 100 m (Scale 3) for elevation. (PDF) [file pone.0063931.s001.pdf]

**Figure S2**

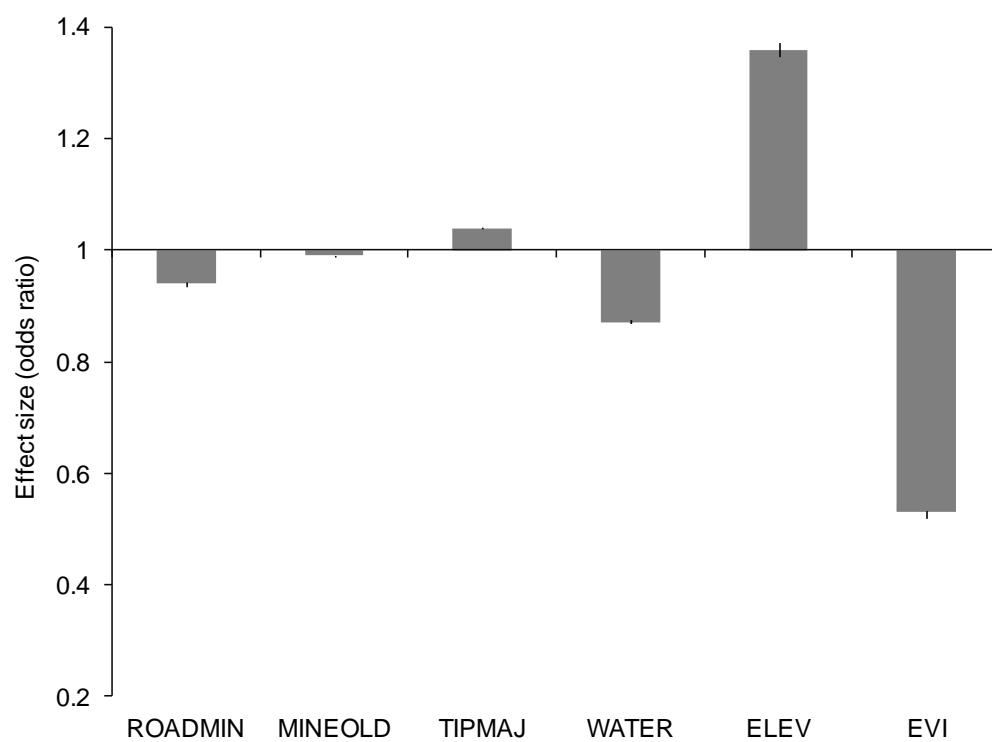

Supplement: Figure S2 — Effect size of continuous predictors of occurrence of dingoes in the ‘all dogs’ model based on the results from the full generalized linear mixed model (GLMM) at a spatial scale of 1 km (Scale 2) for distance predictors and 10 m (Scale 2) for elevation . Odds ratios are provided ±95% confidence intervals (CI). (PDF) [file pone.0063931.s002.pdf]
