# Supplementary material for: Anthropogenic Resource Subsidies Determine Space Use by Australian Arid Zone Dingoes: An Improved Resource Selection Modelling Approach
Source: PLoS One. 2013 May 30;8(5):e63931. doi: 10.1371/journal.pone.0063931 (PMC3667862; doi:10.1371/journal.pone.0063931)
Supplement: Table S1 — Chi-squared (χ2) tests of the deviance of the full models to a null model (no predictors) and mean average prediction for where dingoes were present (prediction – present) and absent (prediction – absent). Df = degrees of freedom. *** = P<0.001. (PDF) [file pone.0063931.s003.pdf]

Table S1

Model	Scale	χ^2	Df	*P*	Prediction – Present	Prediction – Absent
Mine	1	56446	12	***	0.76	0.05
	2	59464	12	***	0.79	0.04
	3	59464	12	***	0.79	0.04
Intermediate	1	14802	11	***	0.27	0.15
	2	16058	11	***	0.28	0.14
	3	16058	11	***	0.28	0.14
Away	1	9297.7	10	***	0.27	0.15
	2	11511	10	***	0.29	0.14
	3	11511	10	***	0.29	0.14
All Dogs	2	52913	8	***	0.33	0.13
