# Supplementary material for: Anthropogenic Resource Subsidies Determine Space Use by Australian Arid Zone Dingoes: An Improved Resource Selection Modelling Approach
Source: PLoS One. 2013 May 30;8(5):e63931. doi: 10.1371/journal.pone.0063931 (PMC3667862; doi:10.1371/journal.pone.0063931)
Supplement: Table S2 — Parameter estimates (β) and standard errors (SE) for continuous predictors included in the full models in the Tanami Desert. Models were fitted with a random intercept following Gillies et al. [10]. *** = P<0.001. (PDF) [file pone.0063931.s004.pdf]

Table S2

	Scale 1 (1 m)			Scale 2 (1 km)			Scale 3 (10 km)		
Predictor	β	SE	*P*	β	SE	*P*	β	SE	*P*
Mine									
Enhanced vegetation index	-0.47	0.02	***	-0.48	0.02	***	-0.48	0.02	***
Elevation	0.04	0.00	***	0.32	0.02	***	3.24	0.21	***
Distance to road (minor)	0.00	0.00	***	-0.65	0.03	***	-6.49	0.33	***
Distance to road (major)	0.00	0.00	***	-0.15	0.01	***	-1.49	0.14	***
Distance to mine (old)	0.00	0.00	***	-0.50	0.01	***	-5.00	0.07	***
Distance to refuse facility (minor)	0.00	0.00	***	-0.45	0.01	***	-4.54	0.11	***
Distance to refuse facility (major)	0.00	0.00	***	-0.70	0.01	***	-7.02	0.13	***
Intermediate									
Enhanced vegetation index	-0.32	0.01	***	-0.32	0.01	***	-0.32	0.01	***
Elevation	-0.05	0.01	***	-0.05	0.01	***	-0.55	0.08	***
Distance to road (minor)	0.00	0.00	***	-0.12	0.00	***	-1.22	0.04	***
Distance to mine (old)	0.00	0.00	***	0.03	0.00	***	0.30	0.01	***
Distance to refuse facility (major)	0.00	0.00	***	0.02	0.00	***	0.19	0.01	***
Distance to water	0.00	0.00	***	-0.12	0.00	***	-1.17	0.02	***
Away									
Enhanced vegetation index	-0.10	0.01	***	-0.18	0.01	***	-0.18	0.01	***
Elevation	0.01	0.00	***	0.19	0.01	***	1.88	0.10	***
Distance to road (minor)	0.00	0.00	***	-0.04	0.00	***	-0.36	0.03	***
Distance to refuse facility (major)	0.00	0.00	***	0.05	0.00	***	0.49	0.01	***
Distance to water	0.00	0.00	***	-0.13	0.00	***	-1.33	0.02	***
All Dogs									
Enhanced vegetation index	-	-	-	-0.64	0.01	***	-	-	-
Elevation	-	-	-	0.31	0.00	***	-	-	-
Distance to road (minor)	-	-	-	-0.06	0.00	***	-	-	-
Distance to mine (old)	-	-	-	-0.01	0.00	***	-	-	-
Distance to refuse facility (major)	-	-	-	0.04	0.00	***	-	-	-
Distance to water	-	-	-	-0.13	0.00	***	-	-	-
